# Rhinometry: Spectrum of nasal profile among Nigerian Africans

**DOI:** 10.1590/S1808-86942011000500009

**Published:** 2015-10-22

**Authors:** Rabiu O. Jimoh, Sulyman B. Alabi, Aremu Shuaib Kayode, Ajao M. Salihu, Olubukola D. Ogidi

**Affiliations:** 1 FMCR (Lecturer/Consultant Surgeon/ Anatomist); 2 FWACS (Senior Lecturer/Consultant Otolaryngologist.); 3 FWACS (Consultant Otolaryngologist. Otolaryngology); 4 (MBBS, MSc Anatomy), Lecturer in Anatomy. E-mail: moyoajao@yahoo.com; 5 (BSC), Anatomist

**Keywords:** evaluation, nasal cartilages, nose, nasal bone, rhinoplasty, nasal septum

## Abstract

**Abstract:**

Nasal parameters measurements are useful in anthropology to distinguish people into racial and ethnic groups.

**Materials and methods:**

A cross-sectional survey among Nigerians aged 18 to 70 years of Nigerian parentage randomly selected at the ENT Clinic of the University of Ilorin teaching hospital (U.I.T.H.), Ilorin, Nigeria without gender discrimination had measurement of their nasal parameters done using a sliding caliper: Nasal height, width, tip protrusion, alar thickness, nasal septal thickness and nares diameter.

**Results:**

105 subjects were seen, the age range 18 to 70 years (mean of 28.63 + 13.06 years). There was 58 males and 47 females with a male/female ratio of 1.2:1. The mean nasal width/height (Nasal index -NI) was 90.7 in males and 88.2 in females. Males had a higher NI compared to female (*p* < 0.03). The commonest type of nasal variability is Type A (70.5%), Platyrrhine nose, Type B (26.7%) especially in females (mesorrhine) and Type C (leptorrhine) (2.8%).

**Conclusions:**

There is significant association between the sex of an individual and type of Nose. Platyrrhine nose, among males and mesorrhine among females, only 2.8% being leptorrhine. The nasal indices were higher in males than in females.

## INTRODUCTION

The nose is an important part in an individual's appearance, hence concerns on the form resulting in increasing demand for rhinoplastic operations^1^. The characteristics and differences of the shapes of the nose have been studied among different ethnic groups^2^. Variables that determine the shape of the nose include race, tribe and environmental climatic conditions, with narrower noses being favored in cold and dry climates and broader noses in warmer, moister ones as a consequence of natural selection in human evolution^3^.

The importance of nasal morphometric parameters is recognized in nasal surgical and medical management^4^.

Nasal index (NI) is one of the important anthropometric parameters for suggesting the race and sex of an individual whose identity is unknown^5^, ^6^, ^7^.

Various studies have been done to classify the nose into platyrrhine (black), mesorrhine (oriental) and leptorrhine (whites) noses^8^,^9^.

The nose is classified as follows: leptorrhine if the nasal index is less than 70, platyrrhine (broad nose)^8^,^9^ if greater than 85 and mesorrhine if between 70 and 85. A study by Ofodile et al, ^6^ among black- Americans classified their noses as Group A African, Group B Afro – Caucasian, Group C Afro – Indian. 44%, 37% and 19% belonged to Group A, B and C respectively.

Oladipo et al.^4^ studied nasal indices (NI) among the major ethnic Groups in southern Nigerian using 1675 subjects in the age range 22 to 30years. The mean NI in his series was 88.6 with the Ijaws having the highest of 96.4, Igbos 94.1 and Yorubas 89.2.

There are various categories of nose on the basis of nasal height, nasal breadth and nasal index. The three categories are commonly accepted^4^. Values of the nasal parameters together with nasal index among African tribes from various geographical regions and ethnic make up are not as readily available as figures from other parts of the world. In Nigeria, Akpa et al.^10^ and Oladipo et al.^4^ respectively did morphometric studies of the nasal parameters among Nigerians of Igbos descent, south eastern, Nigeria and ethnic groups in Southern Nigerian respectively.

This prospective study on nasal rhinometry and NI measurements was done on individuals in Ilorin, North central Nigeria most of whom are of Yoruba ethnic group as found in south western Nigeria, this will provide a baseline data which may be important in rhino-plastic surgery for repair of nasal trauma or cosmesis, anthropological and forensic studies.

## PATIENTS AND METHODS

A cross-sectional survey of volunteer Nigerians between 18 and 70 years old, born in Nigeria with Nigerian parents, without any history of nasal fracture, prior plastic surgery, or any gross facial or nasal deformity were seen at the Otolaryngological clinic of the University of Ilorin teaching hospital, Ilorin, North central Nigeria between January and July 2007. The subjects were randomly selected, of no mixed race. Study design is anthropometric survey (Rhinometry). Ethical clearance was secured from the hospital authority and individual patient consents were also sought.

After giving informed consent, each subject underwent standard facial photography (frontal and lateral views) with 8 mega pixel digital camera so as to use photographic analysis to evaluate:

1 Dorsum shape (D_s_), done by taking the lateral view of the face concentrating on the nose and the shapes were classified as: Straight slope, Concave dorsum and Convex dorsum.
2 Ala lobule prominence (A_LP_) was done by taking frontal and lateral Views of the face to see if the lobule was protruding or if the lateral walls formed a relatively straight slope from the tip to the ala base. It is classified as very prominent, less and least prominent ala lobule.
3 Nasal tip shape (N_TS_) was classified as full and rounded or Pointed.
4 Presence of hump (H).

Then, 5 anthropometric measurements of the nose were obtained using a sliding caliper. Each subject was asked to sit on a low stool and a metal sliding caliper was used to measure the following clinical measurements, termed “rhinometric parameters:

1 Nasal height (N_H_) is the distance from the nasion to the Nasospinale.
2 Maximum Nasal width (N_W_) is the distance from the ala to ala at right angle to the nasal height.
3 Nasal tip protrusion (N_TP_) which is the distance from the nasal septum to the nasal apex.
4 Alar thickness (A_TH_) is the distance from the internal to the External part of each ala.
5 Septal thickness (S_TH_) is the distance taken from the sides of each Septum facing both alae.

Nasal index was calculated as follows: Nasal index = Nasal width / Nasal height (Romo and Abraham, 2003).

Limitations included some subjects unwilling to have their pictures taken and women in purdah who were unwilling to be enlisted.

Precaution taken included cleaning of sliding caliper with 70% alcohol in between patients. Measurements were taken while subject was sitting on a low stool will the head in anatomical position. Errors due to parallax were reduced to the minimum by taking measurements twice and averages taken. Also pictures taken were quickly downloaded into the computer to avoid putting a different face to a different name.

Data were entered on spreadsheets and analyzed using EPI-INFO 2005, 3.3.2 version. Values were expressed as mean ± SD. The level of statistical significance was set at *p*<.05 for all tests.

## RESULTS

105 subjects completed the study, the age range was 18 to 70 years with a mean age of 28.63 + 13.06 years. The average male/female ages were 28.79 + 13.23 and 28.64 + 12.70 respectively. The commonest age range was 18 to 30 years (81%), 51 to 70 years (13%) and 31 to 50 years (11%) as shown in Table 1. There were 58 males and 47 females with male to female ratio of 1.2:1.0.

**Table 1:** Age distribution of respondents.

Age Group	Female	Male	Total
18-30 years	35	46	81
31- 50 years	7	4	11
51-70 years	5	8	13
Total	47	58	105

The average nasal parameters are shown in Table 2 were nasal height (N_H_) which ranged from 5.01 to 5.23cm±0.18 ; Nasal width N_W_ 4.50 to 4.70 cm ±0.24 ; Nasal tip protrusion N_P_ 1.79 – 1.90± 0.09 ; Nasal diameter N_D_ 1.45 to 1.57±0.012 ; Ala thickness A_TH_ 0.69 to 0.83 ± 0.02 and Septal thickness S_TH_ 0.89 to 1.07±0.04. The average nasal index among males was 90.7 (SD 8.1, SE 0.38) and in females it was 88. 2 (SD 8.3, SE 0.47) with a *p*-value of 0.03.

**Table 2:** Average nasal parameters among the age groups (centimeters).

Nasal Parameter	Age Group	Frequency	Average Value
	18-30years	81	5.23
Nh	31-50 years	11	4.96
	51-70 years	13	5.01
	14-30 years	81	4.5
Nw	31-50 years	11	4.6
	51-70 years	13	4.7
	18-30 years	81	1.39
Ntp	31-50 years	11	1.91
	51-70 years	13	1.90
	18-30 years	81	1.45
Nd	31-50 years	11	1.53
	51-70 years	13	1.57
	18-30 years	81	0.69
Ath	31-50 years	11	0.77
	51-70 years	13	0.83
	18-30 years	81	0.87
Sth	31-50 years	11	1.01
	51-70 years	13	1.07

**Figure 1:**
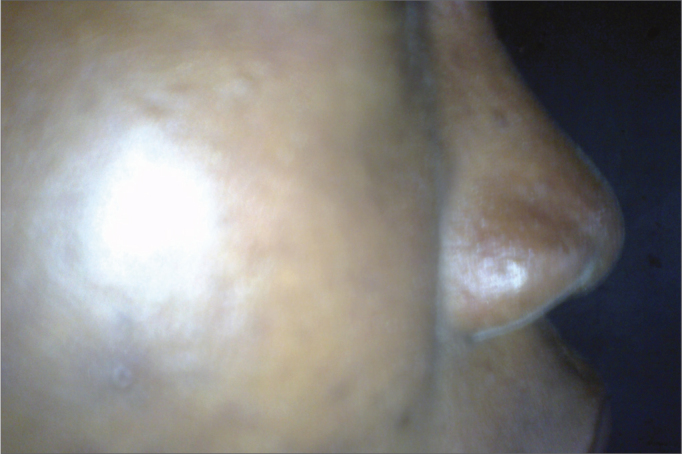
Lateral view of a female respondent with platyrrhine nasal morphology.

**Figure 2:**
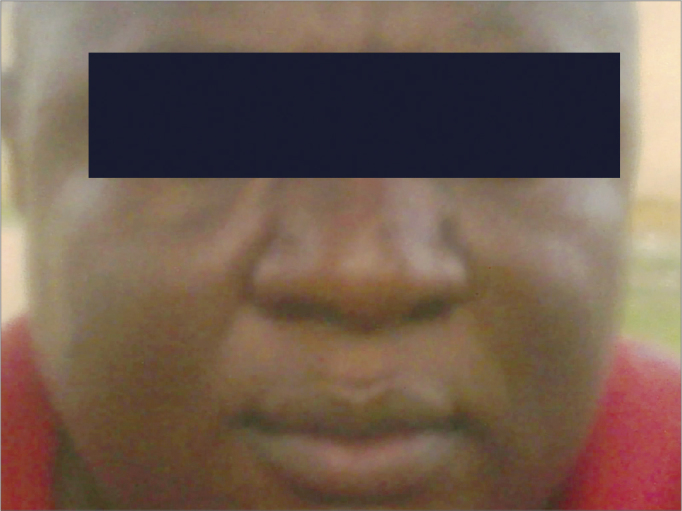
Anterior-Posterior view of a male respondent with platyrrhine nasal morphology

The frequency of nose types among the different age groups in Table 3 and Figures 1 to 2, showed Type A (platyrrhine) was the commonest 73 (69.5%), Type B (mesorrhine) 28 (26.7%) especially in the age group 18 to 30 years (82%) and all the three groups were in the age group and Type C (leptorrhine) being only 4 (3.8%) amongst the age group 18 to 30 years.

**Table 3:** A categorization of nose types among age groups in the study population.

Age	A	B	C	Total
18-30 Years	54	23	4	81
31-50 Years	8	3	-	11
50-70 Years	11	2	-	13
Total	73	28	4	105

Type A: very prominent ala lobule with rounded nasal tip.

Type B: less prominent ala lobule with more defined nasal tip.

Type C: least prominent ala lobule with well designed nasal tip.

The nasal parameter indices were consistently higher in the males compared to the females except in N_T_ to N_W_ index.

## DISCUSSION

The racial and ethnic features of each patient's nose are dependent on the underlying bony and cartilaginous skeletal frameworks together with the skin and the soft tissue envelopes^9^. These features have genetic basis but are also influenced by environmental factors such as trauma, ageing, nutrition and surgery^9^. Environmental climatic condition are believed to influence the shape of the nose with narrower noses being favored in cold and dry climates and broader noses in tropical environment^3^,^11^.

Several studies have reported racial and ethnic differences in nasal index amongst diverse population groups. According to Abraham & Romo^9^ and Risley^11^ respectively, most Western Europeans have leptorrhine nasal morphology with long and narrow nose with a nasal index of 69.9 or less; the Bantus and Bushmen of South Africa as well as indigenous aboriginals in Australia have platyrrhine nose with broad nose with nasal index of 85.0 and above.

The sudroid race and aboriginals have been reported to have a nasal index similar to indigenous Africans south of the Sahara with a nasal index of 85.0 and above i.e.platyrrhine, while the German's nasal index is similar to that of the general Western European's average of nasal index of 71.0 and below (leptorrhine)^11^.

Akpa et al.^10^, from south eastern Nigeria reported that the mean nasal length and width of Nigerian Igbos were 6.22 and 7.26 respectively with a nasal index (NI) of 85.7 while Oladipo et al^4^, from South western Nigeria reported an average NI of 89.2 to 96.4 among three different ethnic groups. From this study, the NI were within the range reported among various workers among indigenous Africans, however males were found to have a higher nasal index compared to the females. This was found to be statistically significant (*p* < 0.03), agreeing with the earlier work of Oladipo et al and Akpan both in southern Nigeria^4^,^10^.

The degree of variability found within the study group of Nigerians showed N_W_ to N_H_ mean for male to be 90.7 and 88.2 in females, still within the range of 60 – 112 similar to that reported by Porter et al.^7^ N_T_ to N_W_ mean is 44.3 for males and 45.7 for females with a range of 31.38 – 59.66. This is consistent with Porter and Olsen et al who worked on analysis of African – American females' noses.^7^

According to a study by Ofodile et al ^6^,among 201 black American, the nose were classified into three groups. Group A which is said to be African, Group B, Afro-Caucasian and group C Afro-Indian.44% belonged to group A, 37% group B and group C were 19%.55% of the African noses had a concave dorsum whereas only 10% of the Afro-Caucasian and 7% of the Afro-Indian groups had a concave dorsum whereas 36% of the Afro-Caucasians had a hump. Anthropometric measurements also revealed that African noses were the shortest and widest, African-Indian the longest and Afro-Caucasians the narrowest as shown in Table 4. The ratios of NT/NH and NT/NW were observe to be highest in the age group of 35-50 as shown in Table 5.

**Table 4:** Nasal measurements among different races.

Nasal Indices	Iranians(F)	Afro-Americans(F)	Korean American(F)	North American(F)	Present Study
NH	57.5	0	51.8	50.6	51
NW	29.16	0	35.5	31.4	46
NTP	34.76	0	0	0	18.7
NI	50.7	79.7	68.5	62.1	89.5
NT/NH	60.5	33.8	0	0	35.3

**Table 5:** Nasal parameter ratios among age groups.

Age Groups (Years)	NT/NH	NT/NW
11-30	34.4(81)	44.6(81)
31-50	38.7(11)	47.0(11)
51-70	38.2(13)	45.5(13)
Total	105	105

The platyrrhine nose constituted the highest, nearly three quarters of the subjects, Type B was second commonest especially among females due to their smaller features. Type C typical among the Europeans was the least, it was found also among the females. This is consistent with previous series^6^, ^7^, ^8^, ^9^. The commonest type of nose, Type A (platyrrhine) is characterized by a very prominent ala lobule and a full and rounded nasal tip typical of African nose followed by Type B which is characterized by a less prominent ala lobule with a more defined nasal tip and the least of was Type C characterized by least prominent ala lobule with a well defined nasal tip. This confirms the existence of ethnic differences among the subjects found in our series.

The flat dorsum with a poorly projected nasal tip in platyrrhine nose is due to lack of bony and cartilaginous supports^9^. The lack of skeletal supports together with thick skin and a prominent subcutaneous fibro- fatty cushion contributes to a poorly projected nasal tip that is amorphous, lacking definition^9^.

## CONCLUSION

In conclusion, there is correlation between the sex of an individual and the type of nose: platyrrhine was commonest in this series as typical of Africans. The average nasal index for males was higher than that of females except nasal tip protrusion – nasal width index showing the existence of sexual dimorphism among the subject. The nasal parameters are within the range identified on blacks south of Sahara Africa with the platyrrhine nose being the commonest with sex differences in nasal index measurements. This will provide a baseline data among Nigerians which will be valuable in physical anthropometry for clinical practice in aesthetic or reconstructive rhinoplastic and also for forensic investigations.

## References

[1] Hormozi AK, Toosi AB. (2008). Rhinometry: an important clinical index for evaluation of the nose before and after rhinoplasty.. Aesthetic Plast Surg..

[2] Hwang TS, Kang HS. (2003). Morphometry of nasal bases and nostrils in Koreans.. An Anat..

[3] Last RJ. (1981). Anatomy applied and Regional;.

[4] Oladipo GS, Olabiyi AO, Oremosu AA, Noronha CC. (2007). Nasal indices among major ethnic groups in southern Nigeria.. Sci Res Essays..

[5] Fransiscus RG, Long JC. (1991). Variation in human nasal height and breadth.. Am J Phys Anthropol..

[6] Ofodile FA, Bokhari FJ, Ellis C. (1993). The black American nose.. Ann Plast Surg..

[7] Porter JP, Olson KL. (2003). Analysis of the African American Female nose.. Plast Reconstr Surg..

[8] Aung SC, Foo CL, Lee ST. (2000). Three dimensional laser scan assessment of the Oriental nose with a new classification of Oriental nasal types.. Br J Plast Surg..

[9] Abraham MT, Romo T. (2006). Rhinoplasty Multiracial, Otolaryngology and facia.. Plast Surg..

[10] Akpa AOC, Ugwu C, Maliki AO, Maliki SO. (2003). Morphometric study of the nasal parameters in Nigerian Igbos.. J Exp Clin Anat..

[11] Risely HH, Crooke W. (1915). The people of India.

